# An exploratory study of associations of physical activity with mental health and work engagement

**DOI:** 10.1186/1471-2458-13-558

**Published:** 2013-06-07

**Authors:** Jantien van Berkel, Karin I Proper, Annelies van Dam, Cécile RL Boot, Paulien M Bongers, Allard J van der Beek

**Affiliations:** 1 Department of Public and Occupational Health - EMGO Institute for Health and Care Research, VU University Medical Center, van der Boechorststraat 7, Amsterdam 1081 BT, The Netherlands; 2 Body@Work, Research Center on Physical Activity, Work and Health, TNO-VU University Medical Centre, Amsterdam, the Netherlands; 3 Department of Work and Employment, TNO Quality of Life, Hoofddorp, the Netherlands

**Keywords:** Work engagement, Physical activity, Accelerometry

## Abstract

**Background:**

Previous studies have found moderate to vigorous physical activity (MVPA) to be associated with a decreased risk of mental disorders. Although the focus in the field of psychology has shifted towards human strengths and optimal functioning, studies examining associations between MVPA and mental health in general (MH) and between MVPA and well-being are scarce. An indicator of work-related well-being is work engagement (WE). The aim of this study was to explore the associations between MVPA and MH, and between MVPA and WE.

**Methods:**

In this study, a total of 257 employees from two research institutes, self-reported their MVPA, MH and level of WE. In addition, a randomly chosen subgroup (n=100) wore an Actigraph accelerometer for a 1-week period to measure their MVPA objectively. Crude and adjusted associations between MVPA and both WE and MH were analyzed using linear regression analyses.

**Results:**

There was no statistically significant association between self-reported MVPA and mental health, resulting from both the crude (b=0.058, 95% CI -0.118 - 0.235) and adjusted analyses (b=0.026; 95% CI -0.158- 0.210), nor between objectively measured MVPA and mental health for both crude and adjusted analyses (b=-0.144; 95% CI -1.315- 1.027*;* b=-0.199; 95% CI 1.417- 1.018 respectively). There was also no significant association between self-reported MVPA and work engagement (crude: b=0.005; 95% CI -0.005-0.016*,* adjusted: b= 0.002; 95% CI -0.010- 0.013), nor between objectively measured MVPA and work engagement (crude: b= 0.012; 95% CI -0.084- 0.060*,* adjusted: b=0.007; 95% CI -0.083-0.069).

**Conclusions:**

Although the beneficial effects of MVPA on the negative side of MH (i.e. mental disorders) have been established in previous studies, this study found no evidence for the beneficial effects of MVPA on positive side of MH (i.e. well-being). The possible difference in how the physical activity-mental health relationship works for negative and positive sides of MH should be considered in future studies.

## Background

Mental health is important for occupational health. Mental disorders are the second most frequent cause of absenteeism from work in Europe, after musculoskeletal disorders [1,2]. Globally, it is one of the leading causes for work disability [1]. However, mental health is not merely the absence of disorders but also comprises well-being [1]. A concept from positive occupational psychology, used as a work-related indicator of well-being, is work engagement (WE). Ouweneel and Schaufeli [3] described WE as work-related happiness, representing a positive affective-cognitive state. WE is negatively associated with burnout, depression, distress and psychosomatic complaints [4,5]. Furthermore, it is considered an antecedent for long-term levels of depression and anxiety symptoms [6] and a predictor of long-term general well-being [7]. In addition, it is positively related to job performance [8] and has shown to be a (negative) predictor of sickness absenteeism from work [5] (Schaufeli et al., 2008).

To date, there is no evidence for effective strategies to improve WE. However, since WE can be equated to happiness at work, it has been hypothesized that evidence based strategies to improve happiness could also be effective to improve WE [3]. Next to psychological strategies to improve happiness, stimulating physical activity is also considered an effective strategy to improve happiness [9]. However, to date, the associations of physical activity and WE remain unexplored.

Research on the physical activity-mental health relationship focuses in particular on mental disorders. Physical activity has been shown to be associated with a decreased risk of mental disorders, such as depression, anxiety and stress [10-13]. Furthermore, Hamer, Stamatakis & Steptoe [14] found a dose–response relationship between physical activity and psychological distress; with a greater risk reduction for activity of higher intensity levels and longer duration. Potential mechanisms for the physical activity- mental health relationship are, for example, biological systems (such as serotonin) and psychological and psychosocial factors (such as improved self-esteem, self-efficacy, perceived behavioral control or cognitive functioning) [15-18]. Physical health could function as a mediator for the physical activity- mental health relationship, although aerobic fitness –often referred to as the golden standard for physical activity- related measures [19] - does not seem to be a plausible mechanism for the physical activity- mental health relationship [10,20].

There is some evidence of a positive association between physical activity and subjective mental well-being outside the work domain. Hamer and Stamatakis [21] found that self-reported moderate to vigorous physical activity (MVPA) was linked to subjective psychological well-being, while objectively measured MVPA was not. Our hypothesis is that the same associations are to be found between MVPA and mental health (MH) and between MVPA and WE, i.e. high MVPA is associated with better MH and higher WE. Hence, the aim of this study was to explore these associations, by both objective measures (accelerometry) and self-reports for MVPA.

## Methods

### Design

This study was conducted as part of a randomized controlled trail (RCT) entitled ”Mindful Vitality In Practice”, of which the study design has been published elsewhere [22]. Briefly, this RCT examines the effect of an intervention aimed at improving WE and energy balance related behaviours (i.e. PA, sedentary and dietary behaviour) among workers. Ethical approval was given by the medical ethics committee of the VU university medical center in Amsterdam, the Netherlands. For this study, data from the baseline measurement were used, which enabled us to use the entire study population and thereby increase power.

### Study population

The study population consisted of workers from two Dutch research institutes. All workers were eligible for participation in this study. However, sick leave for more than 4 weeks and being pregnant for more than 12 weeks at the moment of inclusion were exclusion criteria. The study population consisted of 257 participants.

### Sample size

The sample size of the RCT was based on finding an effect on the primary outcome; work engagement. An effect of a 10% increase in mean score was expected to be relevant and feasible. With a power of 90% and a two-sided alpha of 5%, both groups needed 89 participants. Accounting for a loss to follow-up of 25% over 12 months, each group needed 119 workers at baseline, thus an initial total of 238 participants for the two groups. The sample size calculation has been described more extensively elsewhere [22]. Because of the number of independent variables in the model, this number is expected to be enough to show associations, under the assumption that 10 cases are needed for each independent variable.

### Measurements

#### Work engagement

The Utrecht Work Engagement Scale (UWES) was used to measure work engagement (WE). The UWES is a self-report questionnaire, which measures three aspects of engagement: vigour (6 items), dedication (5 items), and absorption (6 items) [4]*.* Vigour refers to high levels of energy and resilience, the willingness to invest effort, not being easily fatigued, and persistence in the face of difficulties. Dedication refers to deriving a sense of significance from one’s work, feeling enthusiastic and proud about one’s job, and feeling inspired and challenged by it. Absorption refers to being totally and happily immersed in one’s work. Answers were given on a 7-point scale from zero to six, with higher scores representing higher level of work engagement*.* The UWES has shown sufficient internal consistency [4]*.*

### General mental health

General mental health was measured using the corresponding items within the Mental Health Inventory from the RAND-36 [23]. Participants were asked to indicate on a six-point Likert-scale (ranging from constantly to never) for five items how often they felt anxious, depressed, calm, sad and happy during the past four weeks. The items about how often they felt calm and happy were recoded. Further, the items were summed into a rough scale score. This rough scale score was transformed into a final score on a 100 point scale, by dividing the rough scale score minus the minimum rough scale score (which is 5) by the score range (which is 25) and then multiplied by 100 [23]. The final score indicates mental health on a 100 point scale, with low scores representing a negative mental health status over the past four weeks (feeling depressed, sad and anxious) and high scores representing a positive mental health status (feeling calm and happy). The Dutch version of the RAND-36 mental health scale has shown to be sufficiently reliable [23].

### Physical activity

Physical activity was measured both objectively by self-report in a questionnaire and by accelerometry. The results were converted from minutes per week to hours per week by dividing the minutes by 60.

### Self-report

Self-reported physical activity was assessed using the Short Questionnaire to Assess Health Enhancing Physical Activity (SQUASH) [24]. This questionnaire was found to be adequately reliable and valid in a Dutch sample of adult employees [24]. Questions include the frequency and duration of time spent undertaking various intensities of physical activity in leisure time, transport-related activity, work-related activity, and domestic activity. For each of these four domains, participants are required to estimate the number of days a week and duration a day they spent undertaking such activities in a regular week in the past few months.

For the present study, the four domains were not separately analyzed, but summed up into the category ‘MVPA’, in line with Hamar and Stamatakis [21]. First, and then total time spent in this category (MVPA) was calculated. To calculate the mean number of minutes per day spent in moderate (3–6 METs), and vigorous (>6.0 METs) activities, activities were classified into intensity categories using Ainsworth’s compendium of physical activities [25]. Then total time spent in the overall category (MVPA) was calculated by adding these two categories together. A maximum of 6720 minutes per week of activity was kept as a limit for the total possible amount of activity, based on 8 hours of sleep per day.

### Accelerometry

Time spent in MVPA was measured objectively using an accelerometer (Tri-axis Acti trainer activity monitor, Actigraph) and scored and interpreted using Meter Plus version 4.2 software from Santech, Inc. (La Jolla, California). Randomly selected participants of a subgroup (n=100) signed informed consent to wear an accelerometer on the waist during a period of 7 consecutive days. A valid day counts at least 10 hours of wearing time (Ward, Evenson, Vaughn, Rodgers, & Troiano, 2005). Non-wearing periods were determined by a threshold of >30 minutes with 0 counts/minute. Although the best epoch duration to use in adults has not been systematically studied [26], an epoch duration of 60 seconds was used based on previous research [27,28]. The accelerometer objectively determines the cumulative time spent each day in activity at all intensity levels [29]. Calibration of the data to intensity levels was based on cut-off points by validated thresholds [30-32]. The cut-off point for the different intensities of physical activities varies in the literature [33]. For the present study, cut-off points as defined by Freedson et al. [30] were used. For moderate physical activity, >1951-5724 counts per minute was used [30]. For vigorous physical activity, the recommended cut-off point of >5725 counts per minute was used [30,33]. The sum of these two categories formed the category ‘moderate to vigorous PA’ (MVPA). Mean number of wearing days was 6.7. A threshold of at least 3 wearing days was considered a valid week. Total time spent in this category in minutes per week was calculated by summing all valid time periods in that category, divided by the number of valid wearing days (resulting in minutes per day) as the number of wearing days may vary across participants, and consequently multiplied by 7 (resulting in minutes per week).

### Covariates

The analyses of the associations between MVPA and WE, and MVPA and MH were adjusted for socio-demographic variables (e.g. age, gender, and educational level). These variables (age, gender, education, and marital status) were also checked for potential effect modification, (adding interaction terms to the regression model). Additionally, Body Mass Index (BMI) was taken into account because BMI was previously found to be associated with mental health and also with physical activity [34]. For the calculation of BMI, researchers measured the anthropometric variables body weight and height. Height was measured to the nearest 0.1 cm without shoes. Weight was measured to the nearest 0.5 kg in participants wearing indoor clothing and no shoes, after emptying their pockets.

### Statistical analyses

Linear regression analyses were used to examine the associations of MVPA with WE, and MVPA with MH, in SPSS (Chicago, Illinois) version 15.0. Both crude (model 1) and adjusted analyses were performed. Adjusted analysis included the covariates age, gender, educational level, and BMI (model 2). All the analyses were performed for the self-reported and objectively measured data separately.

## Results

An overview of the characteristics of the study population is presented in Table 1. The mean age of the study population was 46 years and two third (67%) of them was female. The large majority (81%) had a high level of education (see Table 1). WE had a mean score of 4.1 (sd=0.8) on a scale from 0 to 6, with higher scores representing more favourable WE. MH had a mean score of 77.0 (sd=13.5), on a scale of 0 to 100, with higher scores representing more favourable MH. The mean Body Mass Index score was 24.7 kg/m^2^. On average, self-reported MVPA was 11.2 hours per week. On average, objectively measured MVPA consisted of 3.6 hours per week. Mean valid days of wearing time of the accelerometer was 6.7 days per week. The subgroup wearing the accelerometer was comparable to the total study population in terms of socio demographics and outcome variables (MH and WE).

**Table 1:** Characteristics of the study population

**Variables**	**Total study population**	**Subgroup wearing accelerometer**
	**(n=257)**	**(n=100)**
**Gender** Female, %	67%	66%
**Age,***mean (sd)*	46 (10)	47 (10)
**Educational level,** High^*^, %	81%	76%
Body Mass Index**,*mean (sd)*	24.7 (3.8)	25.6 (4.0)
Work Engagement (UWES)***,*mean (sd)*	4.0 (0.8)	4.1 (0.8)
Mental Health (RAND-36)***,*mean (sd)*	74.2 (13.5)	77.0 (13.5)
**Self-reported moderate to vigorous physical activity** (hours per week), *mean (sd)*	11.2 (9.4)	11.1 (10.0)
**Objectively measured moderate to vigorous physical activity** (hours per week)*, mean (sd)*	n.a.	3.6 (2.3)

* High educational level: higher vocational education or university.

** Objectively measured.

***Self-reported.

Table 2 shows the results of the linear regression analyses. There was no statistically significant association between self-reported MVPA and mental health, resulting from both the crude (b=0.058, 95% CI -0.118 - 0.235) and adjusted analyses (b=0.026; 95% CI -0.158- 0.210), nor between objectively measured MVPA and mental health for both crude and adjusted analyses (b=-0.144; 95% CI -1.315- 1.027*;* b=-0.199; 95% CI 1.417- 1.018 respectively). There was also no significant association between self-reported MVPA and work engagement (crude: b=0.005; 95% CI -0.005-0.016*,* adjusted: b= 0.002; 95% CI -0.010- 0.013), nor between objectively measured MVPA and work engagement (crude: b= 0.012; 95% CI -0.084- 0.060*,* adjusted: b=0.007; 95% CI -0.083-0.069).

**Table 2:** Results of the linear regression models assessing the associations of moderate to vigorous physical activity with mental health and work engagement

	**Mental Health**		**Work Engagement**	
**Variables**	**Model 1: crude b; 95%CI**	Model 2: adjusted * b; 95%CI	**Model 1: crude b; 95%CI**	Model 2: adjusted * b; 95%CI
**Self-reported (n=257)**				
Moderate to vigorous physical activity	0.058; -0.118 - 0.235	0.026; -0.158- 0.210	0.005; -0.005-0.016	0.002; -0.010- 0.013
**Objectively measured (n=100)**				
Moderate to vigorous physical activity	-0.144; -1.315- 1.027	-0.199; -1.417- 1.018	0.012; -0.084- 0.060	0.007; -0.083-0.069

* Adjusted for age, gender, educational level and BMI.

## Discussion

The aim of this study was to explore the association between MVPA and MH, and between MVPA and WE. Although MVPA was found to reduce the risk for mental disorders in several previous studies, we found no evidence in this study for the aforementioned associations. Hamer & Stamatakis [21] also found no associations between objectively measured MVPA and well-being, although they did find an association between self-reported MVPA and well-being.

Although associations of PA with the negative side of mental health (i.e. mental disorders) were previously found, it could be that the PA-MH relationship works differently for the negative (i.e. mental disorders) and the positive side (i.e. well-being, work engagement) of MH. For example, it could be that more PA is associated with a reduction of the risk of depression, but this does not necessarily imply that it is associated with an increase of the odds of happiness or comparable mental states such as work engagement. This possible difference should be considered when examining potential mechanisms for the PA- MH relationship. Potential psychosocial mechanisms for the PA-MH and PA-WE relationship (for example: PA enlarges self efficacy and self esteem, which could contribute to MH and WE) could be explored in qualitative research, as this provides insight in the nature of this relationship and possible mechanisms.

A possible explanation for the lack of an association between MVPA and WE, might be that WE is work-related to such an extent, that it is not affected by behaviours outside the work domain (e.g. leisure time MVPA). This might explain why previous studies have found associations with work-related factors, such as job demands and resources [5,35], financial returns [36] and job performance [8]. In future studies on associations of behaviours (such as MVPA) and WE, it is recommended to consider the different domains of the PA behaviour. Although this might be the case for WE, this does not explain why there was a lack of associations between MVPA and general MH.

Another explanation for the lack of associations could also be the amount of time spent in MVPA. It is possible that the participants did not perform enough MVPA to show an association with MH or WE. The study population indicated to perceive barriers such as lack of time to engage in leisure time physical activity [22]. When aiming for an increase in physical activity, such barriers should be addressed. Also, it is possible that the data showed no significant results as a consequence of a lack of statistical power. This could be the case for both the objective data, which were available for a subgroup of 100 participants, as for the self-reported data, which were available for the total study population. However, since the b’s were very small, it is considered that greater statistical power would not lead to relevant results.

When comparing the objectively measured data to the subjective data, it appears that in our study, participants tended to overestimate their physical activity. This was also the case in the validation study of the SQUASH: the mean absolute amount spent in each intensity category was consistently higher for the SQUASH than for the accelerometer [24]. This could be due to reporting bias as self-reported physical activity is known to suffer from this type attributable to a combination of social desirability bias and the cognitive challenge associated with estimating frequency and duration of physical activity [37]. In addition, it could be due to some characteristics of the questionnaire; for example one hour of tennis, reported in the SQUASH, is equal to one hour of vigorous activity. An accelerometer does not measure 60 minutes of vigorous activity while playing tennis, but only short bouts of vigorous activity and in-between bouts of moderate or light PA.

### Strengths and limitations

A first strength of this study is, that it is the first study exploring the association between MVPA and MH, and the association between MVPA and WE. Another strength of this study is the use of both self-reported and objective measurements to asses PA. While self-reported PA, through questionnaires, is subject to numerous sources of error, few studies have examined subjective well-being in association with objectively measured PA [21].

Despite the advantages of the objective measurement, the accelerometer has also some limitations. Single waist worn accelerometers are unable to detect specific activity loads, such as load carriage of pulling or pushing and walking on different terrain. Mainly household activities can be substantially underestimated through counts per minute [38]. To classify activity type dual placement, two hip worn or placement around the ankle, should be considered [39]. Moreover, another issue regarding accelerometry data is that activity intensity thresholds, or cut-off points, vary in the literature. Cut-off points should be refined to capture the full range of activity [28,33,39]. Also, cut-off points should be age specific. In the literature on calibration, different thresholds are given for children and adults [40], but no distinction is made in cut-off points for moderate and vigorous for older adults compared to younger adults. However, the calibration study for the cut-off point used in this study was performed with young adults with a mean age of 23 years [30], while the mean age of the subgroup of the study population wearing the accelerometer was considerably higher (47 years).

Finally, it should be taken into account that the sample consisted of mainly higher educated participants in scientific professions. Barkhuizen and Rothmann [41] found that higher educated workers were more engaged than their lower educated colleagues. However, our sample of highly educated workers were averagely engaged, with a slightly higher mean 4.1(SD = 0.8) than the a UWES-17 mean of a Dutch sample (n=2313) of 3.8 (SD=1.1) [4]. Differences in mean levels of engagement between various occupational groups might be significant, but relatively small and they almost never exceed the size of one standard deviation [4]. Thus, these results might be representative for other highly educated workers in scientific professions. Nevertheless, it is not recommended to generalize the results to different professions; it could be argued the relationship between physical activity and work engagement is different for professions that require for example more physical activity at work.

## Conclusion

In summary, the findings of this study show no associations between moderate to vigorous physical activity (MVPA) and mental health (MH), nor between MVPA and work engagement (WE). Although the beneficial effects of MVPA on negative aspects of MH (i.e. mental disorders) have been established in numerous previous studies, this study found no evidence for the beneficial effects of MVPA on positive aspects of MH (i.e. well-being). The possible difference in how the physical activity-mental health relationship works for negative and positive sides of MH (i.e. the working mechanisms) should be considered in future studies.

## Competing interests

The authors declare that they have no competing interests.

## Authors’ contributions

All authors contributed to the conceptual design of the study and intellectual input into the design of this paper. JvB organized data collection, analysed data and drafted the manuscript. AvD wrote a master thesis, of which parts have been used for the manuscript. KIP, CRLB, PMB and AJvdB contributed to the further writing of the manuscript and read and approved the final version of the manuscript. All authors read and approved the final manuscript.

## Pre-publication history

The pre-publication history for this paper can be accessed here:

http://www.biomedcentral.com/1471-2458/13/558/prepub
